# IDSL_MINT: a deep learning framework to predict molecular fingerprints from mass spectra

**DOI:** 10.1186/s13321-024-00804-5

**Published:** 2024-01-18

**Authors:** Sadjad Fakouri Baygi, Dinesh Kumar Barupal

**Affiliations:** Department of Environmental Medicine and Public Health, Icahn School of Medicine at Mount Sinai, CAM Building, 3rd Floor, 17 E 102 St, New York, NY 10029 USA

**Keywords:** Mass spectrometry, Metabolomics, Lipidomics, LipidMaps, Transformer, Molecular fingerprint descriptor, Deep learning, PyTorch

## Abstract

**Abstract:**

The majority of tandem mass spectrometry (MS/MS) spectra in untargeted metabolomics and exposomics studies lack any annotation. Our deep learning framework, Integrated Data Science Laboratory for Metabolomics and Exposomics—**M**ass **INT**erpreter (IDSL_MINT) can translate MS/MS spectra into molecular fingerprint descriptors. IDSL_MINT allows users to leverage the power of the transformer model for mass spectrometry data, similar to the large language models. Models are trained on user-provided reference MS/MS libraries via any customizable molecular fingerprint descriptors. IDSL_MINT was benchmarked using the LipidMaps database and improved the annotation rate of a test study for MS/MS spectra that were not originally annotated using existing mass spectral libraries. IDSL_MINT may improve the overall annotation rates in untargeted metabolomics and exposomics studies. The IDSL_MINT framework and tutorials are available in the GitHub repository at https://github.com/idslme/IDSL_MINT.

**Scientific contribution:**

Structural annotation of MS/MS spectra from untargeted metabolomics and exposomics datasets is a major bottleneck in gaining new biological insights. Machine learning models to convert spectra into molecular fingerprints can help in the annotation process. Here, we present IDSL_MINT, a new, easy-to-use and customizable deep-learning framework to train and utilize new models to predict molecular fingerprints from spectra for the compound annotation workflows.

**Supplementary Information:**

The online version contains supplementary material available at 10.1186/s13321-024-00804-5.

## Introduction

Metabolomics and exposomics fields deal with large volume datasets on the detection and measurement of expected and novel chemical compounds in biological samples [1]. These datasets are mostly generated using untargeted assays employing a gas or liquid chromatography connected to a high-resolution mass spectrometry (HRMS). These instruments can be instructed to fragment chemical compounds in the biospecimens, and the mass to charge ratio and the intensity of those fragments can be recorded in a tandem MS/MS spectrum. In a typical study (n = 100), 2000–3000 unique MS/MS spectra can be collected. These spectra need to be annotated with chemical information such as structure, sub-structure or molecular formula in order to interpret their biological relevance. However, lack of annotations for a majority of the collected MS/MS spectra remains to be a major challenge.

Traditionally, mass spectral libraries available from commercial providers such as NIST or Wiley, in-house, and public resources such as MoNA or GNPS are utilized to annotate the experimental MS/MS spectra with a chemical structure. While this approach is straightforward to use due to the availability of thoroughly benchmarked software such as MS-DIAL, NIST MS Search, Compound Discoverer,and Mass Hunter, a large proportion (> 80%) of MS/MS spectra do not have any hit in these libraries [2, 3].

Machine learning (ML) models can boost the annotation rates. ML models that are trained using curated MS/MS spectra can predict the structure directly from a MS/MS spectrum without the need for searching against mass spectral libraries. For example, CSI:FingerID [4] can predict molecular fingerprint, Spec2Vec [5] can create spectral embeddings, Mass2SMILES [6] can predict SMILES strings, and MSNovelist [7] can predict new structures for a MS/MS spectra. Recently, the large-language models (LLMs) created using the transformer deep learning approach have shown to outperform classical machine learning approaches for the language related tasks such as translation or generating new text. The transformer model uses encoder-decoder structure to map a sequence of tokens from one domain into a different domain, for example translating sentences in English to German. Converting a MS/MS spectrum to molecular descriptor is in a way, a language translation problem, in which token A (m/z and intensity) are translated into token B (descriptors). This idea of token translation has been explored by Spec2Vec [5] to create intermediate embedding, which can be used to search mass spectral or chemical structure libraries (MS2DeepScore [8] and MS2Query [9]). Other individual models such as MS2Mol [10], MS2Prop [11], Mass2SMILES [6] and MSNovelist [7] use the transformer or Long Short-Term Memory (LSTM) architecture to predict chemical descriptors, two-dimensional structure or SMILES notations.

There are two ways to use deep learning in computational mass spectrometry. First to use these individual models. But they have been trained on different training datasets and these models may not be available for a local deployment. They may also not have included specific chemical classes that a user might be interested in. The second approach is to provide an easy-to-use deep learning framework in which a user can train their own models using the MS/MS data that they may have generated for in-house standards or they have access to. For routine chem-informatics tasks, Chemprop [12] is a key example of this second approach to democratize the deep learning methods so anyone who has access to training data and computing power can train different models by optimizing hyperparameters for customized training sets. For example, using Chemprop as a backbone, deep neural network model to predict antibacterial activity properties of > 107 million molecules from the ZINC15 database have led to the discovery of new candidate compounds with antibiotic properties [13]. Similarly, Chemprop [12] has been used in several other contexts to predict pharmacokinetics [14] and molecular properties [15], making it a central resource for mapping chemical-to-function relationships.

**Fig. 1:**
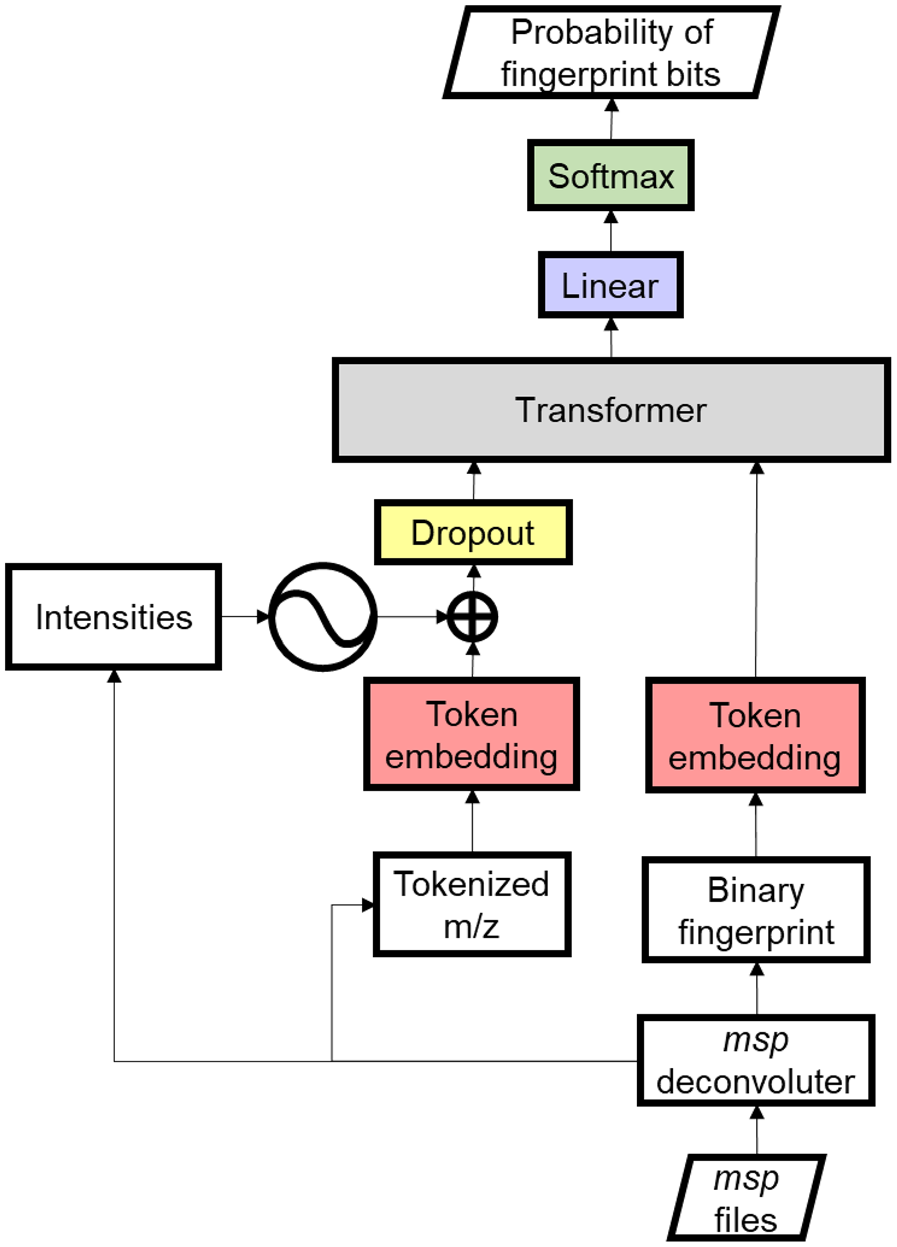
Schematic of MS2Fingerprint model

Inspired by Chemprop [16], to democratize the deep learning in computational mass spectrometry, we have developed IDSL_MINT (https://github.com/idslme/IDSL_MINT), a deep learning mass spectrometry framework, designed to create data-centric models for mass spectrometry applications using transformer models developed by Vaswani et al*.* [17]. IDSL_MINT encompasses modules designed to predict molecular fingerprint descriptors (Fig. 1). We expect that the IDSL_MINT framework will be used to train a diversity of models to predict molecular descriptors from MS/MS spectra.

## Methods

### Modeling framework

IDSL_MINT was developed upon the transformer model architecture originally proposed by Vaswani et al*.* [17] and is implemented within the python PyTorch framework (https://pytorch.org). The IDSL_MINT transformer model architecture for predicting molecular fingerprint is displayed in Figs. 1. IDSL_MINT only takes MS/MS spectra accompanied with precursor *m/z* values in the*.msp* text format. First, *m/z* fragments are tokenized using constant *m/z* interval steps, and intensity values are sorted by the maximum intensity after normalization to the unit sum of the total intensities. Similarly, precursor *m/z* values are tokenized and coupled with spectral entropy values [18] as their pseudo-intensity values when spectral entropy is allowed in the MSP deconvolution step; otherwise, pseudo-intensity values of '2' are utilized. Tokenized precursor *m/z* and its pseudo-intensity are concatenated to the tokenized vectors of *m/z* fragments and fragment intensity values, respectively to create tokenized *m/z* and intensity tensors. Next, tokenized *m/z* tensors are embedded into the transformer model dimension. We hypothesized the intensity of ions in a mass spectrum indicates the abundance of the ions and consequently the importance of the related sub-structure. Therefore, we used a sinusoidal positional encoding similar to Vaswani et al*.* [17] to account for the effects of intensity on the tokenized embedded *m/z* tokens, as presented in Eqs. (1) and (2).

1 $$P{E}_{(pos, 2i)}=sin \left(int.\frac{pos}{{10000}^{\frac{2i}{{d}_{model}}}}\right)$$

2 $$P{E}_{(pos, 2i+1)}=cos \left(int.\frac{pos}{{10000}^{\frac{2i+1}{{d}_{model}}}}\right)$$

where, *PE*, *pos*, *int*, and *d*_*model*_ represent positional encoding tensor, intensity rank order, intensity and model dimension, respectively. The intensity-weighted positional encoding tensor is summed up with the embedded m/z tokens followed by a dropout layer to control potential overfittings. This positional encoding method enabled the utilization of both the precursor *m/z* as molecular mass [11] and the fragment ions. The embedded *m/z* space is converted into the encoder structure of the transformer model. In case, customized binary fingerprint bit locations are not provided within the MSP blocks for training, IDSL_MINT can generate molecular fingerprint descriptors using SMILES or InChI strings for extended connectivity fingerprint (ECFP) using an adjustable radius [19] or MACCS Keys [20] fingerprint types which are widely recognized in the field of cheminformatics [21]. The fingerprint bit locations are submitted to the transformer decoder in an ascending order, but no positional adjustment was used on fingerprint bit locations. The output of the decoder structure of the transformer was fed into a linear layer followed by a SoftMax layer to calculate the probability of the fingerprint bit locations (Fig. 1). The cross-entropy LOSS function is used with adjustable label smoothing followed by an Adam optimizer. A beam search inference is used to ensure detection of the most probable neural network trajectory and to prevent autoregressive shortcuts in transformers. Tanimoto coefficient is calculated to find similarity between predicted and the true positive fingerprints in the accuracy plots.

### Model training setup

IDSL_MINT code is publicly available at https://github.com/idslme/IDSL_MINT. IDSL_MINT was developed using Python 3.10 and implemented with PyTorch 2.0. IDSL_MINT can be executed via a simple and easy to modify YAML configuration files using a simple command “*MINT_workflow –yaml path/to/yaml/file*” in a Linux terminal. Typically, users should specify the criteria for MS/MS spectra input, molecular fingerprint type, transformer model settings, parameters for the cross-entropy LOSS function, and configurations for the Adam optimization strategy. The framework works only in the Linux environment. The model weights are exported at the directory outlined in the configuration file *(.yaml*), for the minimal LOSS value achieved during the training phase. To ensure model reproducibility, we suggest preserving training YAML files as configuration log entries to utilize consistent model parameters in the predictive stage.

### Spectral file for the test model training

IDSL.CSA [2] provided fragmentation spectra databases (FSDBs) for publicly available spectral databases (https://zenodo.org/record/7530397) in positive and negative modes [2]. A subset of LipidMaps spectra from these FSDBs originated from MassBank of North America (MoNA) and Global Natural Product Social Molecular Networking (GNPS) libraries was converted into standard*.msp* files after excluding in-*silico* predicted MSP blocks using LipidBlast method [23]. Classes of lipids were derived from LipidMaps database [24] via matching corresponding InChIKey14 values.

### Test data set

To benchmark the IDSL_MINT framework, we have used a publicly available untargeted metabolomics study from the Metabolomics WorkBench (accession: ST002044) [25]. IDSL.IPA [26] was used for peak-picking and peak alignment followed by MS/MS peak detection using IDSL.CSA [2]. Unique spectra across the entire study were collected in separate MS/MS spectra files in the*.msp* text format for electrospray ionization (ESI) positive and negative modes.*.msp* text files and parameter spreadsheet used to annotate the unique spectra are provided at ( https://zenodo.org/records/8339614) [27] (file#CSA_spreadsheet.zip) for positive and negative modes.

### Model parameters for the test study

In the training step for the test study, only MS/MS spectra were used that had 90% of *m/z* fragments within a range of 50–1000 using 0.01 Da intervals. The extended connectivity fingerprint with a radius of 2 (ECFP2) was utilized to transform MS/MS mass spectrometry data. The MS2Fingerprint models include 4 hidden encoder and decoder layers and 2 attention heads (80 × 10^6^ parameters including parameters of the embedding layer). A Google Colab notebook showing the training and prediction parameters are provided at https://drive.google.com/file/d/1IFWTeeZ_I4tbQ-y6MEvSkQ0hb6vqited/view?usp=sharing. Model training curves are shown in Additional file 1: Figure S1.

## Results

**Fig. 2:**
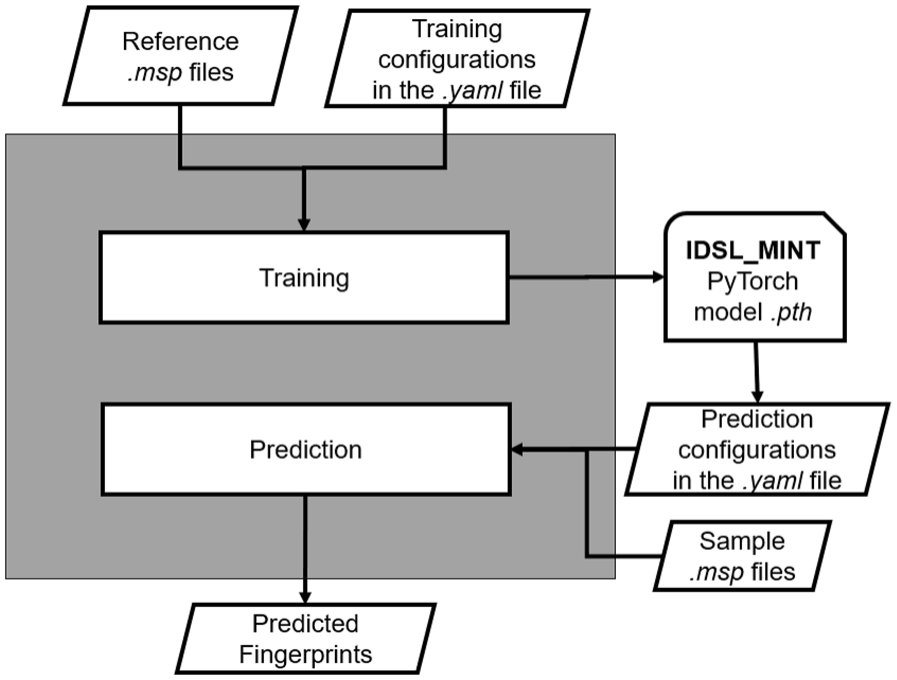
Flowchart of the IDSL_MINT workflow

We have developed IDSL_MINT, a cheminformatics deep learning framework, to predict molecular fingerprint descriptors from MS/MS fragmentation spectra. The framework enabled easy and straightforward training of predictive models from different mass spectral libraries, in a similar fashion to the widely accepted Chemprop approach [16] which can create different models for predicting physical–chemical properties from chemical structures. The framework utilizes a transformer architecture [17] in PyTorch to translate *m/z* values into a fingerprint bit vector. The required input for training the model is only a reference spectra library in the NIST standard*.msp* text format with or without fingerprint data and a model configuration text file (*.yaml*). Figure 2 illustrates the overview of the input and output data in the framework.

As an alternative to interact with complex python scripts, we have provided well documented *yaml* configuration files for selecting model parameters. Similar to Chemprop [16], IDSL_MINT is directly run in a Linux terminal using a simple command (See method). We provided a Google Colaboratory notebook (https://colab.research.google.com/drive/16A-Hw6S_04nxlopp7yefZkVB5Aakcodu) to demonstrate the application of the IDSL_MINT framework without any local installation. Users can train a new model on these notebooks using the test data or they can use their own input data for creating customized models. GPUs and CPUs are both supported by IDSL_MINT. The codebase and the documentation are provided at https://github.com/idslme/IDSL_MINT to install IDSL_MINT in a local Linux server.

We showcase the application of IDSL_MINT for a publicly available untargeted metabolomics study (ST002044) from the Metabolomics WorkBench database. First, we processed the raw data for this study to extract MS/MS spectra in the NIST.msp format that can be used as the model evaluation set. The IDSL.CSA R package [2] was used to extract 3,386 and 1,901 unique MS/MS spectra in ESI^+^ and ESI^−^ for this study [27] (file#1 csa_spreadsheet.zip). Next, we obtained the coverage of spectra annotation by mass spectral similarity searches. IDSL.CSA can annotate 638 (out of 3386) and 299 (out of 1901) MS/MS peaks in positive and negative modes by matching the spectra against NIST 20 and LipidBlast [23] libraries [27] (file#1 csa_spreadsheet.zip).

**Fig. 3:**
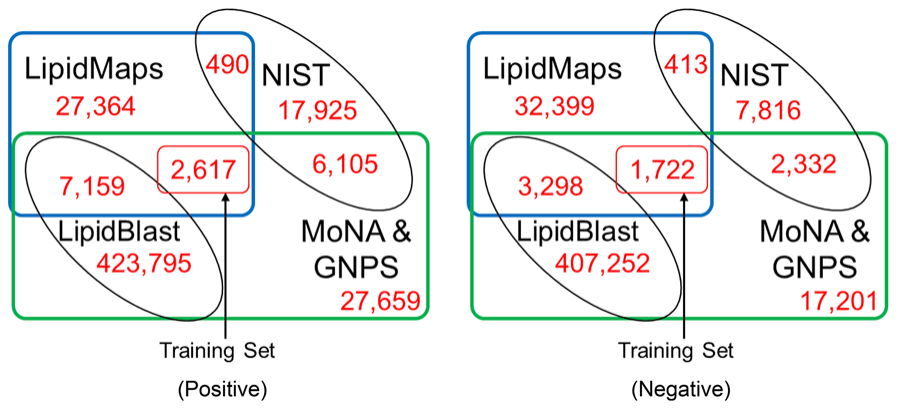
Careful compilation of the training set for the case study. Unique InChIKey14 was used to compute set overlaps. Source data file is provided at Zenodo entry [27] (file#3 venn.zip)

Next, we carefully compiled the training dataset for the case study (ST002044) to ensure a high validity of the resulting model (Fig. 3). Spectra from MoNA and GNPS public libraries in positive and negative modes were used separately to create training sets. We excluded the spectra related to compounds that were covered in the LipidBlast (in-*silico*) [23] and NIST 20 libraries because we used these two libraries for annotating the experimental MS/MS spectra in the case study. Our case study training set included 6.96% and 4.58% of all the compounds in the LipidMaps database in positive and negative modes, respectively because most of the lipid compounds did not have publicly available MS/MS spectra. Lipid classification ontology was not covered in the MS/MS databases, so we obtained it from the LipidMaps structure SDF file in the PubChem database. Training data has 8 lipid classes and 6 classes having at least 50 spectra (Table 1). The most over-represented lipid classes were Polyketides (PK) and Fatty Acyls (FA) and under-represented classes were Glycerolipids (GL) and Saccharolipids (SL). Extracted MS/MS LipidMaps spectra in MoNA and GNPS public libraries, and lipid classes from PubChem database were provided in the*.msp* text format at Zenodo entry [27] (file#2 lipidmaps_msp.zip/). Despite the small size training set, we were able to train a useful model with practical prediction accuracies for an untargeted metabolomics experiment, as highlighted in the following sections.

**Table 1:** Frequency of lipid in each class in the training data set

Lipid class	Negative	Positive
Polyketides (PK)	794	1019
Fatty Acyls (FA)	791	1294
Glycerophospholipids (GP)	339	202
Sterol Lipids (ST)	289	516
Prenol Lipids (PR)	141	438
Sphingolipids (SP)	80	54
Glycerolipids (GL)	5	64
Saccharolipids (SL)	2	1

Next, we trained two deep learning models (ESI^+^ and ESI^−^) using our newly created IDSL.MINT framework. The*.yaml* file and input*.msp* files are provided at Zenodo entry [27](file#4 ms2fp_yaml.zip). The training in ESI^+^ and ESI^−^ took 70 and 45 h, respectively on 35 CPU processing threads.

Next, we evaluated the overall accuracy and the accuracy by lipid classes for the test study ST002044. Since not all lipid classes were equally represented in the training set, we expected that the model prediction accuracy would vary across the classes. Overall accuracy for the case study was 0.354 and 0.351 for positive and negative modes, respectively, where true positive being the top hit candidate when the predicted fingerprint was searched against the LipidMaps database. In terms of lipids classes, Glycerophospholipids (GP) showed an accuracy ≥ 0.50 whereas Prenol Lipids (PR) showed a really low accuracy ≤ 0.20 in both positive and negative modes (Table 2 and file#5 ms2fp_st002044_prediction.zip at [27]). These variations can be attributed to the coverage of these lipid classes in the training dataset. For the case study, IDSL.CSA yielded annotations for 536 lipid compounds for both ESI modes (Table 2). Subsequent matching of the predicted fingerprints using a Tanimoto similarity threshold of ≥ 0.6 resulted with additional 238 lipid annotations in both ESI modes. Furthermore, a spectral entropy score of ≥ 0.75 was found to align with fingerprint matches characterized by a Tanimoto similarity threshold of ≥ 0.7 shown in Table 2. Notably, within the Glycerophospholipids (GP) lipid class, the median Tanimoto similarity stands a score of 1 across 178 annotations with a spectral entropy score of ≥ 0.75 in positive mode. We noted that 71 (ESI^+^) and 96 (ESI^−^) annotations were not part of the training set, suggesting the use of IDSL_MINT for improving annotations beyond mass spectral libraries. The results of all annotated MS/MS spectra in ST002044 by fingerprint matches with Tanimoto similarity ≥ 0.5 against LipidMaps and IDSL.CSA match is provided at Zenodo entry [27](file#6 ms2fp_lipidmaps.zip and file#7 ms2fp_idsl.csa.zip).

**Table 2:** Tanimoto coefficients of ECFP2 fingerprints for the ST002044 study. Fingerprint matching assisted in adding more annotations

Mode	Lipid categories	LipidMaps (Entire top hits)			Library match after spectral entropy ≥ 0.75)			IDSL_MINT new high-confidence annotations (Tanimoto similarity ≥ 0.6)		
		Count	Mean	Median	Count	Mean	Median	Count	Mean	Median
Positive mode	Fatty Acyls (FA)	592	0.254 ± 0.270	0.140	80	0.672 ± 0.330	0.772	24	0.831 ± 0.121	0.857
	Prenol Lipids (PR)	182	0.181 ± 0.158	0.138	4	0.736 ± 0.008	0.731	3	1.000 ± 0.000	1.000
	Polyketides (PK)	233	0.205 ± 0.122	0.181	–	–	–	3	0.881 ± 0.068	0.836
	Sphingolipids (SP)	56	0.326 ± 0.282	0.192	16	0.571 ± 0.381	0.798	–	–	–
	Glycerophospholipids (GP)	724	0.543 ± 0.350	0.540	178	0.916 ± 0.154	1.000	85	0.838 ± 0.111	0.839
	Glycerolipids (GL)	60	0.561 ± 0.368	0.556	35	0.772 ± 0.262	0.875	–	–	–
	Sterol Lipids (ST)	295	0.302 ± 0.272	0.168	65	0.669 ± 0.232	0.673	12	0.805 ± 0.119	0.756
Negative mode	Fatty Acyls (FA)	437	0.233 ± 0.198	0.175	8	0.556 ± 0.301	0.533	22	0.782 ± 0.106	0.757
	Prenol Lipids (PR)	65	0.139 ± 0.029	0.145	–	–	–	–	–	–
	Polyketides (PK)	175	0.183 ± 0.118	0.166	–	–	–	3	0.783 ± 0.155	0.701
	Sphingolipids (SP)	0	–	–	–	–	–	–	–	–
	Glycerophospholipids (GP)	371	0.671 ± 0.247	0.704	150	0.739 ± 0.182	0.714	85	0.837 ± 0.140	0.849
	Glycerolipids (GL)	11	0.335 ± 0.046	0.339	–	–	–	–	–	–
	Sterol Lipids (ST)	121	0.165 ± 0.096	0.144	–	–	–	1	0.887 ± 0.000	0.886

## Discussions

We have developed a deep learning framework for computational mass spectrometry in metabolomics and exposomics to train user-defined models to predict structure and molecular fingerprints from tandem mass (MS/MS) spectra. An application of this framework is presented to annotate MS/MS spectra that did not have any hits in the existing reference mass spectral libraries. It can be suggested that a combined use of both mass spectral library searches and deep learning models can improve the annotation rate in lipidomics, metabolomics and exposomics fields. As we highlighted that our work is inspired by Chemprop [12], to democratize deep learning in computational mass spectrometry, in which users can train custom models by different training datasets. An example is shown as a lipid structure prediction model trained only on the lipid MS/MS spectra because a lipid extraction method was used using the sample preparation. This framework also enables open science where deep learning models created using publicly available MS/MS spectra can be made available without any restrictions.

Transformer models have revolutionized the field of natural language processing (NLP), particularly in transforming between two spaces of sequences of tokens similar to natural language structures [17]. The transformer model consists of encoder and decoder structures which include self-attention mechanisms, feed-forward neural networks, and normalization layers. The ability to tokenize raw mass spectrometry data (*m/z* values) and molecular fingerprint bit locations allow for the adoption of this well-developed model architecture in the field of computational mass spectrometry.

The key objective in using deep learning models for mass spectrometry data processing is to transform mass spectrometry *m/z* values into a more applicable information space such as molecular fingerprint descriptors [28]. This conversion enables utilizing MS/MS spectra in several known cheminformatics approaches such as Quantitative Structure–Activity Relationship (QSAR) modeling. The predicted fingerprints can prioritize the annotated peaks for further structure elucidation workflows which are resource-demanding in mass spectrometry-based projects. Only a limited number of high-quality mass spectral signatures should be prioritized for further in-*silico* analyses and laboratory experiments. QSAR models built by molecular fingerprint descriptors can be utilized to directly predict physical–chemical properties without fully knowing the two-dimensional structure. This was achieved by MS2Prop tool to predict quantitative assessment of drug-likeness (QED) [29], and synthetic accessibility [30].

One of the key messages of our work is that the accuracy of these deep learning models varies by chemical classes. That can be explained by limited training data for them. If a class is not well-represented in the training set of an existing model, then we need to acquire the standards, collect the MS/MS spectra for them and train a new model. This situation is regularly observed by the untargeted metabolomics and exposomics community. It is also recognized in the cheminformatics field that not every class has training data [31–33], an issue known as the domain of applicability [34]. IDSL_MINT will enable straight-forward training of such new models, in a similar way Chemprop [16] supports the QASR modeling. Users should note that a model might perform well for one class, and thus can be used reliably for that specific class, for example the case study works for lipid classes Glycerophospholipids (GP). We have shown for the case study, the 238 additional spectra could be annotated by this new approach; however, the reliable annotations were for the classes with higher accuracies. Our results underscore the need to expand the training data for these under-represented classes in the public libraries such as MoNA and GNPS databases.

Putting together the training and prediction architecture requires setting up complex python scripts, but IDSL_MINT has minimized these efforts to almost no scripting by accepting all the parameters in well-documented configuration files in a simple text format (*.yaml*). This idea of using a*.yaml* file was also adopted from the Chemprop framework [16], and it makes it very straightforward to train a deep learning model for MS/MS spectra. We also use the input spectra in*.msp* text format which is a commonly used format in computational mass spectrometry, meaning that IDSL_MINT can be easily integrated with other workflows which handle mass spectra in this format. For example, by using only IDSL.CSA [2] and IDSL_MINT, a user can extract and annotate MS/MS spectra by mass spectral similarity and the deep learning based predictive modeling.

Since there are many molecular fingerprints available in cheminformatics and new ones can be created for a collection of chemical structures, IDSL_MINT supports any type of user-provided fingerprint as long as it is in the binary vector format. This is different from existing tools SIRIUS 4 [35] and CSI:FingerID [4] which use a set of fixed molecular fingerprints. However, it should be noted that searching the predicted fingerprints against massive databases such as PubChem may require re-calculations of the custom fingerprints.

The main limitation of this work was that our training set was constrained to only the experimental LipidMaps library which was around < 10% of total LipidMaps compounds. However, we have achieved practical accuracies for a number of lipid classes. The transformer architecture can be expanded to include neutral losses from precursor and *m/z* differences, which may also improve the accuracy of the model. Several advanced formats of the transformer architecture have been developed to train large language models by NLP researchers. Those advances can be tested while translating *m/z* tokens into molecular fingerprints tokens.

The presented IDSL_MINT test model also has several limitations. First, the results of this model could not be compared against that of existing fingerprint prediction models such as CSI:FingerID [4]. Second, the model was created for a lipidomics dataset, so it cannot be generalized to other metabolomics datasets. Lastly, we did not test different molecular fingerprint types while building the model. However, it should be noted that the test model was created only to show the utility of the IDSL_MINT framework, and follow-up investigations are required to increase the impact in reference to these limitations. We also recommend that training datasets used as input for the IDSL_MINT framework are submitted to a public data repository such as Zenodo ( https://zenodo.org/) with a proper version control and data provenance records so the results by different modelling frameworks or models can be logically compared.”

### Supplementary Information

**Additional file 1: Figure S1.** Training curves for the lipid annotation model

## Data Availability

Project name: IDSL_MINT, Project home page: https://github.com/idslme/IDSL_MINT. Operating system(s): Linux. Programming language: Python. Other requirements: None. License: MIT. Any restrictions to use by non-academics: None. Data and results are available at https://zenodo.org/record/8339614

## References

[1] Schrimpe-Rutledge AC (2016). Untargeted metabolomics strategies-challenges and emerging directions. J Am Soc Mass Spectrom.

[2] Baygi SF, Kumar Y, Barupal DK (2023). IDSL.CSA: composite spectra analysis for chemical annotation of untargeted metabolomics datasets. Anal Chem.

[3] Domingo-Almenara X (2018). Annotation: a computational solution for streamlining metabolomics analysis. Anal Chem.

[4] Duhrkop K (2015). Searching molecular structure databases with tandem mass spectra using CSI:FingerID. Proc Natl Acad Sci U S A.

[5] Huber F (2021). Spec2Vec: Improved mass spectral similarity scoring through learning of structural relationships. PLoS Comput Biol.

[6] Elser, D., F. Huber, and E. Gaquerel, *Mass2SMILES: deep learning based fast prediction of structures and functional groups directly from high-resolution MS/MS spectra.* bioRxiv, 2023: p. 2023.07. 06.547963

[7] Stravs MA (2022). MSNovelist: de novo structure generation from mass spectra. Nat Methods.

[8] Huber F (2021). MS2DeepScore: a novel deep learning similarity measure to compare tandem mass spectra. J Cheminform.

[9] de Jonge NF (2023). MS2Query: reliable and scalable MS(2) mass spectra-based analogue search. Nature Communication.

[10] Butler, T., et al., *MS2Mol: A transformer model for illuminating dark chemical space from mass spectra.* 2023

[11] Voronov, G., et al., *MS2Prop: A machine learning model that directly predicts chemical properties from mass spectrometry data for novel compounds.* bioRxiv, 2022: p. 2022.10. 09.511482

[12] Yang K (2019). Analyzing learned molecular representations for property prediction. J Chem Inf Model.

[13] Stokes JM (2020). A deep learning approach to antibiotic discovery. Cell.

[14] Stoyanova R (2023). Computational predictions of nonclinical pharmacokinetics at the drug design stage. J Chem Inf Model.

[15] Liu C (2023). ABT-MPNN: an atom-bond transformer-based message-passing neural network for molecular property prediction. J Cheminform.

[16] Heid, E., et al., *Chemprop: A Machine Learning Package for Chemical Property Prediction.* 2023 10.1021/acs.jcim.3c01250 PMC10777403 38147829

[17] Vaswani A (2017). Attention is all you need. Advances in Neural Information Processing Systems.

[18] Li Y (2021). Spectral entropy outperforms MS/MS dot product similarity for small-molecule compound identification. Nat Methods.

[19] Rogers D, Hahn M (2010). Extended-connectivity fingerprints. J Chem Inf Model.

[20] Yongye AB (2011). Consensus models of activity landscapes with multiple chemical, conformer, and property representations. J Chem Inf Model.

[21] Xie L (2020). Improvement of prediction performance with conjoint molecular fingerprint in deep learning. Front Pharmacol.

[22] Schwaller P (2021). Mapping the space of chemical reactions using attention-based neural networks. Nat Mach Intell.

[23] Kind T (2014). LipidBlast templates as flexible tools for creating new in-silico tandem mass spectral libraries. Anal Chem.

[24] Fahy E (2007). LIPID MAPS online tools for lipid research. Nucleic Acids Res.

[25] Baygi SF (2022). IDSLUFA Assigns high-confidence molecular formula annotations for untargeted LC/HRMS data sets in metabolomics and exposomics. Anal Chem.

[26] Fakouri-Baygi S, Kumar Y, Barupal DK (2022). IDSL.IPA characterizes the organic chemical space in untargeted LC/HRMS data sets. J Proteome Res.

[27] Barupal, S.F.B.D.K., *Data and results for the IDSL.MINT publication*, in *Zenodo*. 2023.

[28] Ji H (2020). Predicting a molecular fingerprint from an electron ionization mass spectrum with deep neural networks. Anal Chem.

[29] Bickerton GR (2012). Quantifying the chemical beauty of drugs. Nat Chem.

[30] Ertl P, Schuffenhauer A (2009). Estimation of synthetic accessibility score of drug-like molecules based on molecular complexity and fragment contributions. J Cheminform.

[31] Lo Y-C (2018). Machine learning in chemoinformatics and drug discovery. Drug Discovery Today.

[32] Chen K (2023). MetaRF: attention-based random forest for reaction yield prediction with a few trails. J Cheminform.

[33] Colby SM (2019). ISiCLE: a quantum chemistry pipeline for establishing in silico collision cross section libraries. Anal Chem.

[34] Sutton C (2020). Identifying domains of applicability of machine learning models for materials science. Nat Commun.

[35] Duhrkop K (2019). SIRIUS 4: a rapid tool for turning tandem mass spectra into metabolite structure information. Nat Methods.

