# Supplementary material for: IDSL_MINT: a deep learning framework to predict molecular fingerprints from mass spectra
Source: J Cheminform. 2024 Jan 18;16:8. doi: 10.1186/s13321-024-00804-5 (PMC10797927; doi:10.1186/s13321-024-00804-5)
Supplement: Supplementary file 1 — Additional file 1: Figure S1. Training curves for the lipid annotation model [file 13321_2024_804_MOESM1_ESM.docx]

**IDSL_MINT: A Deep Learning Framework to Predict Chemical Structures from Mass Spectra**

Sadjad Fakouri Baygi, Dinesh Kumar Barupal*

Department of Environmental Medicine and Public Health, Icahn School of Medicine at Mount Sinai, New York, NY, 10029, USA

* Corresponding author: Address: CAM Building, 3rd floor, 17 E 102nd St, New York, NY 10029, Email: dinesh.barupal@mssm.edu

**Table of Content:**

**Figure S1.** Training curves for the lipid annotation model


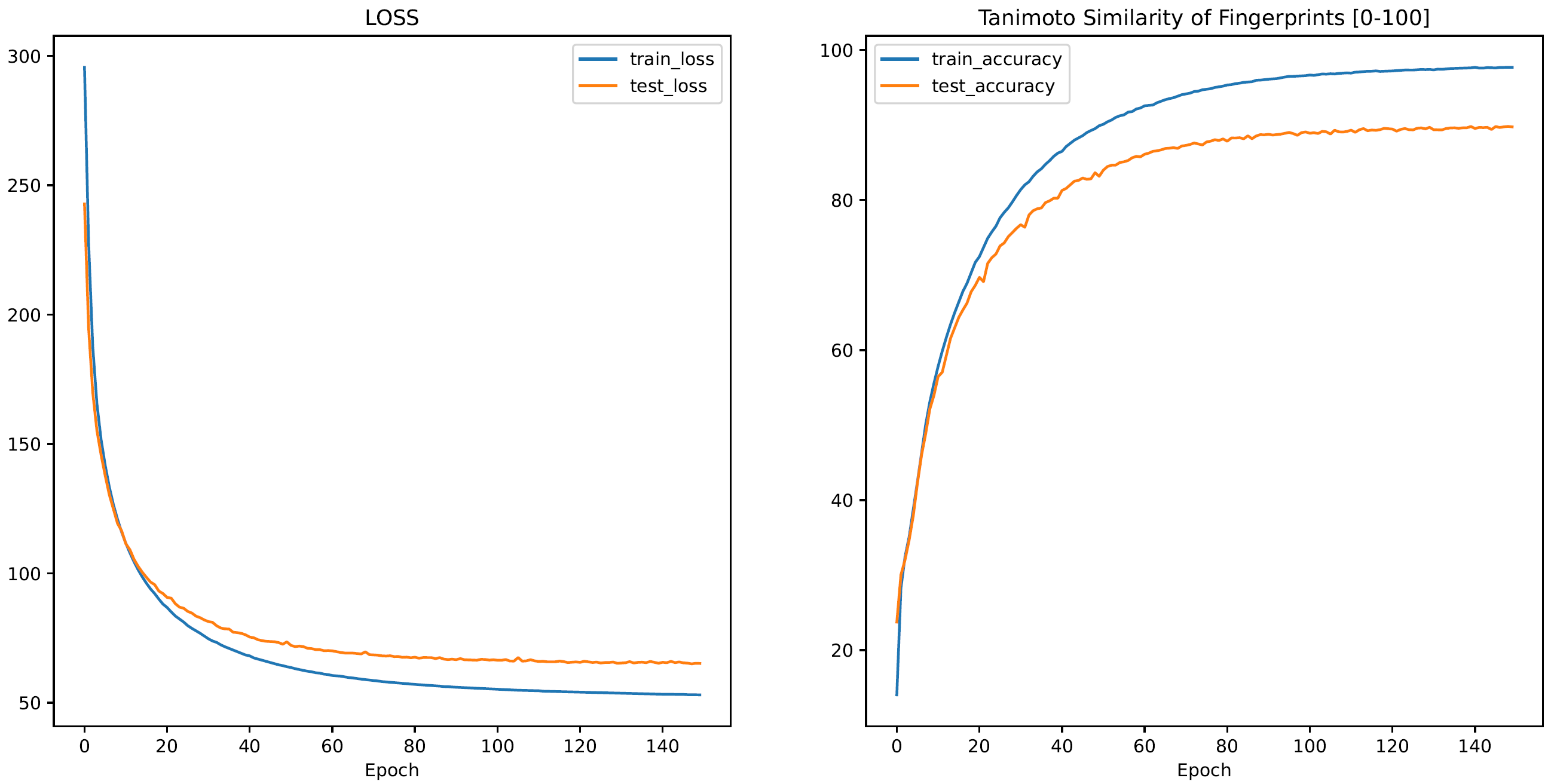

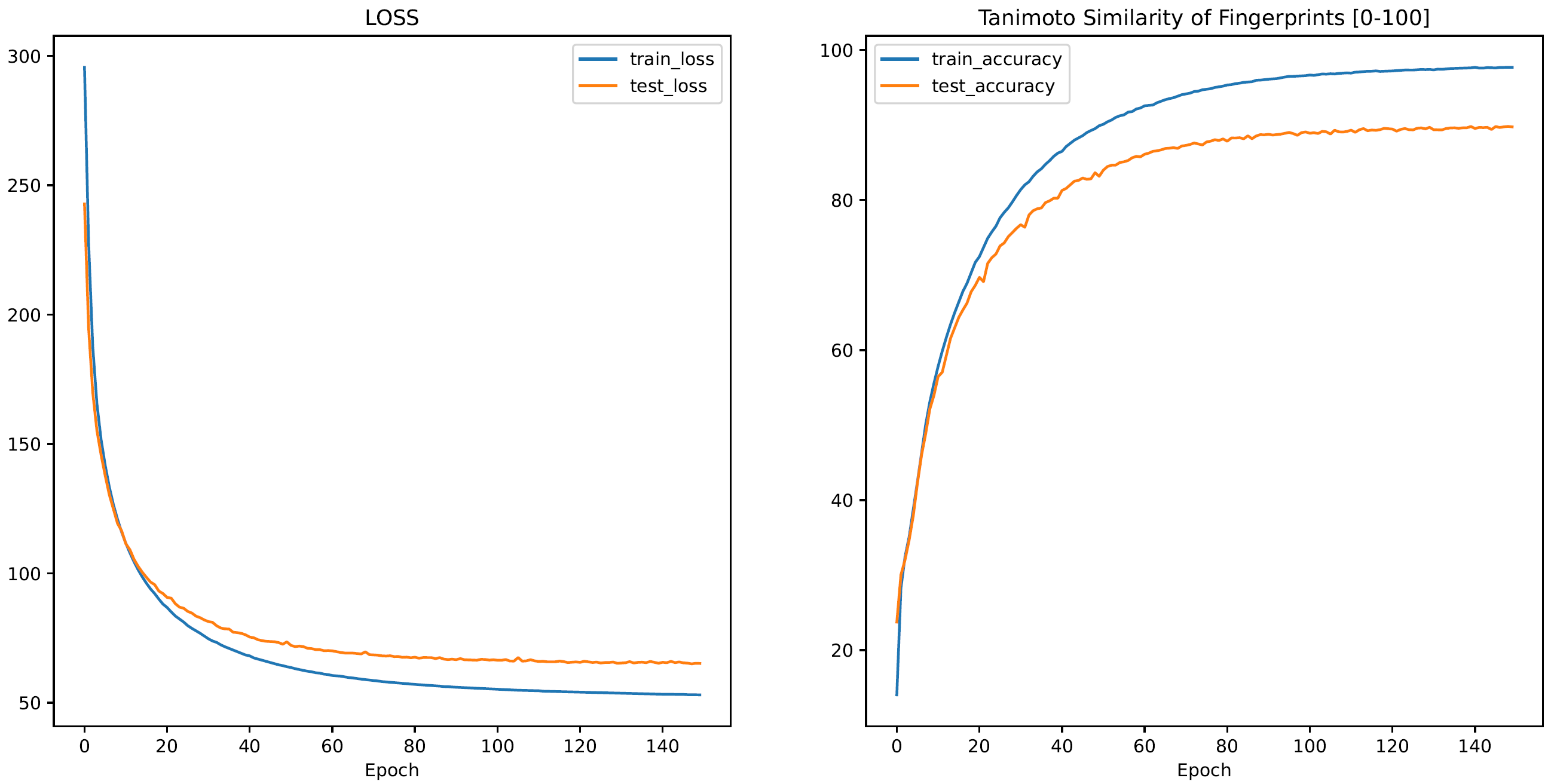


Figure S.1.

ESI negative mode

ESI positive mode
